# Nutrition Knowledge of Caregivers Influences Feeding Practices and Nutritional Status of Children 2 to 5 Years Old in Sekhukhune District, South Africa

**DOI:** 10.4314/ejhs.v32i1.12

**Published:** 2022-01

**Authors:** Tubatse Tiny Motebejana, Cebisa Noxolo Nesamvuni, Xikombiso Mbhenyane

**Affiliations:** 1 Nutrition Department, School of Health Sciences, University of Venda, Thohoyandou, South Africa, and Limpopo Provincial Department of Health in South Africa; tinnymotebejane@gmail.com; 2 Nutrition Department, School of Health Sciences, University of Venda, Thohoyandou, South Africa; cebisa.nesamvuni@univen.ac.za; 3 Human Nutrition Division, Faculty of Medicine and Health Sciences, Stellenbosch University, Tygerberg, 7505, PO Box 241, Cape Town, 8000, South Africa; xgm@sun.ac.za

**Keywords:** Caregivers, nutrition knowledge, feeding practices

## Abstract

**Background:**

The aim of this study was to determine caregivers' nutrition knowledge and its influence on feeding practices and nutritional status on young children.

**Methods:**

A cross-sectional survey was conducted in 120 caregiver-child pairs. A validated questionnaire was used to collect caregivers' nutrition knowledge and feeding practices. Nutritional status was estimated using anthropometric measurements. Z-scores were computed using WHO Antro software. Chi-square test was used to determine the association between caregivers' nutrition knowledge and feeding practices. Significance was set at p <0.05.

**Results:**

Forty three percent of caregivers reported health professionals as their source of nutrition information. Almost all children (94.2%) were breastfed at one stage in their lives, with 62.5% introduced to solid foods before six months. Maize meal porridge (87.5%) and bread (54.2%) were consumed daily by most of the children, while 48.3% consumed meat and meat products three to four times per week. One in eight children consumed fruits daily and 5.8% vegetables daily. Forty one percent of children were stunted. Family income showed positive correlation with the nutritional status of children (weight-for-age R = 0.207, p < .05; height-for-age R°=°0.203*, p°= .026). An association was observed between the duration of breastfeeding and alternative food, to milk (R = 0.302**, p = .001), amount of fruit consumed daily (R = 0.197*, p = .031) and number of meals consumed daily (R = 0.284**, p = .048). Conclusion: Nutrition knowledge and feeding practices of the caregivers were not satisfactory.

## Introduction

Nearly half of all deaths in children under the age of five years are attributable to undernutrition. This translates into the unnecessary loss of approximately three million young lives a year (1). UNICEF further suggests that this number will continue to rise unless strategies are put in place to manage malnutrition (2).

Child survival, growth, and development depend not only on food intake and health, but also on care and the caregiver's behaviour (3). One of the components of the caregiver's behaviour is the feeding of young children (4). WHO recommends that infants should be exclusively breastfed for the first six months of life to achieve optimal growth? Furthermore, infants should receive nutritionally adequate and safe complementary foods, while continuing to breastfeed for up to two years or beyond (5). When exclusive breastfeeding is not practiced, the consequence can contribute to a high prevalence of malnutrition (5).

Delaying the introduction of complementary foods beyond the age of 26 weeks is associated with the risk of nutritional deficiency, particularly in low-income populations, and such delays may be associated with an increased risk for disorders related with the immune system (6). Infants who are offered a wide variety of vegetables in a complementary feeding period may be more accepting of vegetables and fruits. Studies have also indicated that seeking a variety of foods at age two to three years was a predictor of the same behaviour until early in adult life, highlighting the importance of establishing a varied diet in infancy (4,7).

Imdad *et al.* (8) have shown that the caregivers' level of nutritional knowledge is a predictor of feeding practices. Lack of nutritional knowledge on child feeding among caregivers contributes significantly to poor dietary practices of children under five years of age (9; 10). The UNICEF (11) programme report, recommended the need to strengthen the quality of counselling given to mothers and caregivers, and the importance of appropriate behavioural change communication to other family and community decision-makers.

Several nutrition interventions programmes have been launched, targeting young children in South Africa, and include nutrition and health education, promotion of exclusive breastfeeding, growth monitoring and promotion, and implementation of the infant and young child feeding programme. The South African Paediatric Food-Based Dietary Guidelines were also developed to provide a tool for nutrition education. South African Demographic Health Survey (SADHS) (12) observed that at national level, 27% of children under age 5 were stunted, 3% wasted, 6% underweight and 13% overweight while stunting was 22% in Limpopo province. The aim of this study was to determine nutrition knowledge of caregivers and its influence on feeding practices and nutritional status of young children.

## Materials and Methods

The study design was a cross-sectional survey with an analytical component, carried out in households of the caregivers in Makhuduthamaga local municipality in South Africa. Convenient sampling was used to select four villages, while systematic sampling was used to select households with children 2 to 5 years old. On arrival at the village, the first household with qualifying participants was identified, thereafter every fifth household in the same street. If there would be no child, the next household was identified. The final sample was 120 children-caregiver's pairs.

The questionnaire was developed based on literature relating to the caregivers' nutrition knowledge and feeding practices. A pilot study was done to test for content validity, reliability and feasibility. Content validity was also enhanced by having the expert input during the development of the questionnaire. The questionnaire was researcher administered using local language (*Sesotho sa Leboa*) in the area. Anthropometric measurements taken included weight, height and mid-upper-arm circumference (MUAC) of the children as well as weight and height of the caregivers using standard procedures (12).

**Ethical statement**: Ethical approval was granted by the University of Venda ethics committee (ethical clearance, 03.09.2006). Permission to conduct research in the villages was granted by the municipal manager of Makhuduthamaga (03.27.2006) and traditional leaders in the selected villages. Written informed consent was obtained from the caregivers after the aims and the objectives of the study, as well as the procedures of data collection, were explained in detail. Anonymity and confidentiality were maintained by using codes. Participation was voluntary and all participants were informed that they had the right to withdraw from the study at any time without suffering any adverse consequence.

**Data analysis**: WHO Antro was used to compute Z-scores. Data was captured in excel and exported to SPSS version 21 for analysis. Descriptive and inferential statistics were utilised. The descriptive statistics that were used included frequency distribution, mean, standard deviation, ranges, and percentiles. Chi-square was used to identify the association among sociodemographic data, mothers' nutrition knowledge, the feeding practices and nutritional status. The significance level was set at p < .05.

## Results

**Characteristics of the children and caregivers**: The sample consisted of 120 child-caregiver pairs. The majority (70.2%,) of the caregivers were the biological mothers of the children, 66.7% were between the ages of 19 to 35 years, 55.8% were single parents, while 70% had secondary education, 85% were unemployed. See Table 1A.

**Table 1A:** Demographic and household characteristics **(n=120)**

Variable	Frequency	%
**Age ranges**		
16–18 years	1	0.8
19–25 years	35	29.2
26–35 years	45	37.5
Above 35 years	39	32.5
**Marital status**		
Single	67	55.8
Marriage	39	32.5
Separated	1	0.8
Live together	7	5.8
Widowed	6	5.0
**Education level**		
None	14	11.7
Primary	17	14.1
Secondary	84	70.0
Tertiary	5	4.2
**Employment status**		
Employed (Full time)	6	5.0
Unemployed	102	85.0
Retired	7	5.8
Student	3	2.5
Volunteer	2	1.7
**Caregivers' relation to the children**		
Biological mother	85	70.2
Other	35	28.9
**Family size**		
2–3	3	2.5
4–6	51	42.5
>6	66	55.0
**Children below 12 years**		
1–2	58	48.3
3–4	47	39.2
>4	15	12.5
**Number of people working per household**		
None	44	36.7
One	55	45.8
2–3	19	15.8
>3	2	1.7
**Household monthly income**		
< R500	10	8.3
R600–R1000	15	12.5
R1100–R2000	41	34.2
R2100–R3000	36	30.0
R3100–R5000	13	10.8
>R5000	5	4.2
**Source of Nutrition knowledge**		
None	50	41.7
Health professional	52	43.3
Family member	8	6.7
Radio	8	6.6
Newspaper/magazines/book	6	5.0
Formal school	5	4.2
Community health worker	3	2.5
Television	2	1.7
Crèche	1	0.8

The study comprised of 120 children aged two to five years, 54.2% (65) males and 45.8% (55) females. The age distribution was 41% (50) for 36 to 48 months, 37.2% (45) from 24 to 36 months, and 20.7% (25) from 49 to 60 months. The mean and standard deviations of children's age were 3.33 ± 0.76 years. Almost all (94.2%) children received government child support grant. Table 1 provides the anthropometric characteristics of the children. No differences were observed between male and female children, or between age groups. Table 1B also shows the nutritional status of the children as classified by the WHO standards. Most children were stunted (41.7%), 32.5% at risk of stunting while 18.3% were overweight. With respect to the BMI, 30.8% of caregivers were overweight, 30.8% obese, while 0.8% were underweight.

**Table 1B:** Anthropometric data of the children and caregivers

Anthropometry of children Variable	n = 120		
	Total	Female (n=55)	Male (n=65)

	Mean (± SD)	Mean (± SD)	Mean (± SD)
Age (years)	3.33 ± 0.76	3.33 ± 0.76	3.34 ± 0.77
Birth weight (kg)	3.18 ± 0.44	3.23 ± 0.41	3.14 ± 0.46
Birth height (cm)	50.31 ± 2.62	50.28 ± 2.85	50.33 ± 2.43
Birth head circumference (cm)	34.89 ± 1.18	35.04 ± 1.25	34.76 ± 1.12
Weight (kg)	13.88 ± 2.08	13.63 ± 2.04	14.08 ± 2.09
Height (cm)	90.52 ± 7.56	90.54 ± 7.48	90.50 ± 7.68
WHZ	0.78 ± 1.09	0.65 ± 1.19	0.89 ± 0.99
HAZ	-1.89 ± 1.3	-1.73 ± 1.26	2.04 ± 1.45
WAZ	-0.56 ± 0.97	-0.55 ± 0.93	0.56 ± 1.01
BAZ	1.00 ± 1.19	0.79 ± 1.29	1.18 ± 1.07
MUAC (cm)	15.7 ± 1.12	15.58 ± 1.11	15.59 ± 1.13
MUACZ	-0.023 ± 0.88	-0.21 ± 0.87	2.45 ± 0.89
**Nutritional Status classification** **Children** (n=120)			
Stunted (HAZ < -2 SD)	50 (41.7%)	23 (41.8%)	27 (41.5%)
Risk of stunting (HAZ -1 to -2 SD)	39 (32.5%)	16 (29.1%)	23 (35.4%)
Normal height	31 (25.8%)	16 (29.1%)	15 (23.1%)
Underweight (WAZ < -2 SD)	1 (0.8%)	1 (1.8%)	0%
Overweight WHZ (> +2 SD)	22 (18.3%)	9 (16.4%)	13 (20.0%)
Normal weight	55(45.8%)	28(50.9%)	27(41.5%)
**Caregivers**	n= 120		
Underweight (BMI < 18.50 kg/m^2^)	1 (0.8%)	1 (0.8%)	0%
Normal (18.50 to 24.99 kg/m^2^)	45 (37.5%)	45 (37.5%)	0%
Overweight (BMI 25 to 29.99 kg/m^2^)	37 (30.9%)	37 (30.9%)	0%
Obese (≥ 30 kg/m^2^)	37 (30.8%)	37 (30.8%)	0%

WHZ: weight-for-height z score, HAZ: height-for-age z score, BMI: body mass index, WAZ: weight-for-age z score, BAZ: body mass index-for-age z score, MUAC: mid-upper arm circumference, MUACZ: mid-upper arm circumference z score.

**Nutrition knowledge of caregivers**: Caregivers who reported not to have any source of nutrition education were 41.7%, while 43.3% reported health professionals as the source of nutrition education. Media and family members were also cited as sources of nutrition education by some of the caregivers (Table 1A).

Only 35% of the caregivers stated breastfeeding for 24 months and beyond as the recommended duration of breastfeeding. Most of the caregivers (82.5%) fed their children starchy foods. While the majority (82.5%) reported that they did not know the amount of milk needed per day for children two to five years, with only 7.5% of the caregivers reporting two cups of milk per day. See Table 2.

**Table 2:** Caregivers' knowledge on types of food and feeding of children (=120)

Knowledge item	Frequency	Percent
**Best food for children <6 months**		
Breastmilk only	28	23.3
Breastmilk and other foods	48	40.0
Infant formula only	4	3.3
Infant formula and other foods	21	17.5
Don't know	19	15.8
**Recommended breastfeeding duration**		
6–12 months	10	8.3
12–18 months	41	34.2
19–23 months	4	3.3
24 months and above	42	35.0
Do not know	23	19.2
**Alternative foods to porridge**		
Rice, bread or samp	99	82.5
Cabbage or pumpkin	3	2.5
Don't know	18	15
**Alternative foods to meat**		
Legumes, meat products, fish, soya soup	33	27.5
Spinach	25	20.8%
Potatoes	27	22.5
Both legumes and vegetables	1	0.8
None of the above	1	0.8
All of the above	3	2.5
Don't know	30	25.0
**Milk requirement for children 2–5 years**		
½–1 cup	10	8.3
2 cups	9	7.5
One litre	2	1.7
Don't know	99	82.5
**Number of fruits to be eaten by children 2–5 years**		
½ cup	1	0.8
1–1 ½ cup	6	5.0
2–3 cups	18	15.0
4–5 cups	6	5.0
Don't know	89	74.2
**Amount of vegetables eaten per day by the** **children 2–5 years**		
None	1	0.8
1–2 tablespoons	6	5.0
¼ cup	5	4.2
⅓–½ cup	2	1.7
1–1 ½ cups	5	4.2
Small bowl (300 ml)	1	0.8
Occasionally	1	0.8
Don't know	99	82.5
**Number of meals per day for a child**		
Once	0	0
Twice	8	6.7
≥3 times and more	85	70.8
Don't know	27	22.5
**Feeding modality**		
Feed themselves	111	92.5
Fed by the mother	7	5.8
Both the child and the mother	2	1.7
**Duration of breastfeeding**		
<3 months	4	3.3
3–12 months	17	14.2
13–18 months	54	45.0
19–24 months	34	28.3
>24 months	3	2.5
Don't know	1	0.8
N/A	7	5.8
**Age at which solids were introduced**		
<6 month	57	47.5
≥6 months	22	18.3
Not given	41	34.2

Similarly, 74.2% reported that they do not know the amount of fruit recommended per day for children aged two to five years. Those who said it was 1-1½ cup of fruits were only 5.0%. When asked about the number of vegetables recommended per day for a child of two to five years, only 14.2% reported an amount of 1-1½ cups. Most of the caregivers (70.8%) mentioned that the child should be given meals three times and more per day, while 22.5% of the caregivers did not know. See Table 2.

**Feeding practices of children**: The majority of the caregivers (70%) reported that they gave the children three or more meals per day. Most of the children (92.5%) were not assisted during mealtimes. See Table 1. More than two-thirds of the children (94.2%) were breastfed at one stage in their lives. Forty five percent of children were breastfed up to 13 to 18 months, followed by those children (28.3%) who were breastfed up to 19 to 24 months. Almost half of the children (47.5%) were given infant formula while they were still less than six months, 18.3% at more six months while 34.2% were never given formula milk. Some of the caregivers used bottles to give their children formula milk, while others added it to cereal. Only 2.5% used either a cup or spoon. It was found that most of the caregivers (62.5%) introduced solid foods before six months (Table 2).

Maize meal soft porridge was reported as the main solid food introduced first to the children (66.7%), followed by infant cereals (25%). Bottle feeding was the main method of feeding (Table 3).

**Table 3:** Method used to give formula milk to the children (n=120)

Variables	Frequency	%
**Method of giving infant formula**		
Bottle feeding	51	42.5
Added in cereal	23	19.2
Spoon feeding	2	1.7
Both spoon and bottle feeding	2	1.7
Cup feeding	1	0.8
N/A*	41	34.2
**Initiation age of complementary foods**		
<6 months	75	62.5
≥ 6 months	42	35.0
Don't know	3	2.5
**Types of complementary food first introduced to the children.**		
Maize meal soft porridge	80	66.7
Soft porridge, and other foods	2	1.7
Infant cereal	30	25.0
Infant cereals and ready to eat bottled food	1	0.8
Ready to eat bottled baby food	6	5.0
Don't know	1	0.8

* N/A refers to children who were never given formula milk.

The results in Table 4 showed that maize meal porridge (87.5%) and bread (54.2%) were consumed daily by most of the children. Majority (65.0%) of the children were given potatoes three to four times per week. Full cream milk powder was consumed daily by only 20% of the children. Meat and meat products were consumed three to four times per week by almost half of the children (48.3%). Peanut butter was eaten daily by 5.8% of children, while beans were consumed occasionally by 36.6%, and once or twice a week by 24.2% of the children.

**Table 4:** Food frequency consumption by the children

Food items	Percentage consumption frequency (=120)				
	
	5–7x/week	3–4x/week	1–2x/week	Occasionally	Never
**Cereals/starches**					
Stiff porridge	92.5	5.8	0.0	0.8	0.8
Soft porridge	25.0	17.5	10.0	6.7	40.8
Breakfast cereal e.g., corn flakes	5.8	5.8	3.3	17.6	67.5
Rice	0.8	25.8	40.8	31.7	0.8
Bread	55.0	26.7	10.0	7.5	0.8
Samp	0.0	4.2	18.3	39.2	38.3
Potatoes	1.7	65.0	14.2	11.6	7.5
**Dairy products**					
Eggs	6.7	35.8	11.7	31.6	14.2
Fresh milk	10.8	15.0	8.3	15.0	50.8
Full cream Milk powder e.g., Nespray	20.0	10.0	2.5	9.2	58.3
Cheese	0.0	0.8	0.8	18.4	80.0
**Animal foods**					
Polony/Viennas	2.5	14.2	9.2	43.4	30.7
Mopani worms	0.8	2.5	3.3	19.1	74.2
Chicken	10.9	48.3	16.7	23.3	0.8
Chicken feet and heads	3.3	56.7	15.0	10.8	14.2
Chicken Livers	0.0	3.3	4.2	3.3	89.2
Chicken offal	0.0	31.7	19.2	17.5	31.7
Pork	0.0	2.5	0.0	3.3	94.2
Fish	0.8	18.3	18.3	43.3	19.2
**Legumes**					
Beans	2.5	20.0	24.2	36.6	16.7
Soya products	2.5	20.0	14.2	15.8	47.5
Peanuts	0.8	6.7	4.2	15.0	73.3
Peanut butter	11.6	16.7	14.2	34.7	22.5
**Fruit**					
Apple	0.8	30.8	25.0	30.8	12.5
Avocado	0.0	12.5	13.3	47.5	26.7
Banana	2.5	31.7	24.2	30.0	11.7
Fruit juice, concentrated	9.2	27.5	18.3	29.9	15.0
Grapes	0.0	0.8	0.8	55.0	43.3
Guava	1.7	12.5	7.5	49.1	29.2
Mango	0.0	1.7	0.8	58.4	39.2
Naartjie	0.8	2.5	2.5	20.0	74.2
Orange	5.8	40.8	20.0	27.5	5.8
Pawpaw	0.0	0.0	3.3	26.6	70.0
Peaches	0.8	3.3	2.5	74.1	19.2
Pear	0.0	6.7	6.7	14.9	71.7
**Vegetables**					
Cabbage/spinach/ Traditional vegetable	1.1	24.7	26.1	31.7	16.4
Pumpkin/carrot/ Beetroot	0.0	4.2	22.7	40.3	32.8
Sweet potatoes	0.8	2.5	5.8	53.4	37.5
Tomatoes	20.0	59.2	10.0	5.0	5.8
**Miscellaneous**					
Fat cakes	0.0	12.5	9.2	39.1	39.2
Cold drinks	2.5	12.5	16.7	57.5	10.8
Sweets	10	25.8	20.8	31.6	11.7
Potatoes crisps/ maize/corn chips snacks	21.7	42.5	16.7	15.9	3.3
Biscuits	9.2	35.8	24.2	21.7	9.2
Ice cream	0.0	0.8	2.5	27.5	69.2
Margarine	13.4	16.7	9.2	30.1	30.8
Jam	3.3	7.5	5.8	21.7	61.7
Yoghurt	5.8	16.7	13.3	50.0	14.2

* Occasional consumption frequency refers to foods not eaten often, it includes once per month, once in 3 months. or only eaten during functions, or seasonally.

Consumption of fruits and vegetables was found to be very low, as only one in eight children ate fruit daily, and 5.8% of the children ate vegetables daily. See Table 4. Most of the children (56.7%) ate vegetables two to three days per week, while 40% of the children ate fruit two to three days per week. Concentrated juice was reported to be consumed daily by 5% of the children. The results show that most of the children 35% were given potatoes/maize/corn snack chips, sweets (23%), and biscuits (20%) and 12% given fruit, as snacks. See Table 4. The top ten foods that were mostly given to the children were stiff maize meal porridge (98.3%), bread (81.7%), tomatoes (79.2%), potatoes (66.7%), potatoes/maize/corn snack chips (64.2%), chicken heads and feet (60%), chicken (59.2%), oranges (46.6%), biscuits (45%), and eggs (41.7%). These foods are from categories: starch, protein, vegetable, fruit, and snack thus, including most the nutrient categories. The frequency varied from daily to once a week.

**Relationship between sociodemographic data, caregivers' nutrition knowledge, feeding practices, and nutritional status of children**: Table 5 shows the association between nutritional status of children and selected variables. HAZ was positively associated with the source of household income, as well as household monthly income (*p* < .05). Source of income was also associated with weight-for-age (R = 0.207, *p* =.024) and height-for-age (R = 0.203*, *p* = .026). In addition, positive association was observed between height-for-age and range of household income (R = 0.234*, *p* = .010).

**Table 5:** Associations among sociodemographic data, caregivers' nutrition knowledge, feeding practices and nutritional status of children (n = 120 unless specified)

Variable	Statistic	WHZ	HAZ	WAZ	MUACZ
**SOCIODEMOGRAPHIC DATA**					
Marital status	R	-0.041	0.086	0.037	-0.017
	***p***	.660	.348	.692	.853
Level of Education	R	0.080	-0.099	-0.010	0.033
	*p*	.383	.284	.918	.724
Employment status	R	-0.084	-0.046	-0.103	-0.147
	*p*	.360	-.617	.262	.110
Source of income	R	0.047	**0.203** *	**0.207** *	0.063
	*P*	.613	**.026**	**0.024**	.496
Range of Household income	R	-.0058	**0.234** *	0.153	-0.015
	*p*	.530	**0.10**	.095	.871
Child support grant	R	0.017	-0.048	0.054	-0.079
	*p*	.855	-.600	.558	.394
Number of children below 12	R	-0.063	-0.039	-0.079	-0.102
years	*p*	.493	.673	.394	.269
**NUTRITION KNOWLEDGE**				
Best food for < 6 months old	R	0.064	0.079	0.124	0.078
	*P*	.486	.391	.178	.400
Duration of breastfeeding	R	-0.142	0.145	0.000	0.197*
	*P*	.121	.115	.999	.031
Substitute for porridge	R	-0.128	0.056	0.157	-0.124
	*P*	.163	.546	.087	.177
Alternative for meat	R	- 0.092	0.167	0.062	0.001
	*P*	.318	.068	.503	.988
Amount of milk	R	- 0.052	0.016	- 0.027	0.116
	*P*	.575	.862	.773	.208
Alternative for milk	R	-0.113	-0.031	-0.122	-0.108
	*P*	.220	.738	.185	-.242
Amount of fruits to eat daily	R	0.059	- 0.045	0.011	-0.030
	*P*	.522	.623	.903	.742
Amount of vegetables to eat	R	-0.154	0.077	-0.053	0.065
daily	*P*	.093	.404	.564	.480
Amount of water	R	0.282**	**0.190** *	-0.157	- 0.094
	*P*	**.002**	**.038**	.086	.308
Number of meals to eat daily	R	- 0.008	- 0.050	- 0.045	0.103
	*P*	.928	.585	.623	.261
**FEEDING PRACTICES**					
Status of child breastfed	R	0.074	-0.101	- 0.025	- 0.029
	*P*	.422	.274	.784	.753
Duration of breastfeeding	R	0.075	- 0.099	- 0.023	-0.034
	*P*	.416	.282	.803	.715
Giving of infant formula	R	- 0.066	- 0.097	-0.131	0.015
	*P*	.471	.293	.153	.868
Age of introducing infant	R	- 0.097	-0.032	-0.105	- 0.009
formula	*P*	.294	-.728	.254	.922
Method of giving infant	R	- 0.094	-0.030	-0.102	- 0.007
formula	*P*	.305	.745	.270	.935
Time of solids introduction	R	0.082	-0.039	0.036	-0.051
	*P*	.373	.673	.695	.578
Food given 1st	R	0.080	-0.011	0.064	0.072
	*P*	.383	.909	.486	.432
Number of meals given	R	0.031	- 0.003	0.020	-0.155
	*P*	.735	.972	.829	.091

R = correlation coefficient

** p < .01

* p < .05

WHZ: Weight-for-height z scores, HAZ: Height-for-age z scores, WAZ: Weight-for-age z scores, MUACZ: Mid upper arm circumference z scores.

WAZ, WHZ, HAZ and MUAC Z-scores were analysed for association with caregivers' nutrition knowledge. Positive association was observed between caregivers' nutrition knowledge on the amount of water to be taken by the child per day with wasting (R = 0.282**, *p* = .002) and stunting (R = 0.190*, *p* = .038).

WAZ, WHZ, HAZ and MUAC Z-scores were also analysed for association with caregivers' feeding practices. There was no positive association observed between nutritional status of children and caregivers' feeding practices in this study. However, an inverse association was observed between WAZ and the type of milk used in children's tea (R = 0-.221*, *p* = .015).

Furthermore, there was no association between the children's anthropometric variables and the caregivers' BMI.

## Discussion

**Underweight**: The prevalence of underweight was 0.8% which indicates a low prevalence of malnutrition. The findings are lower as compared to the SADHS (12) which reported 6% underweight. Another study reported that the prevalence of underweight among children younger than 10 years of age decreased from 2005 to 2013 in Limpopo to 9.7%, and 8.8% at national level (13).

**Stunting**: Stunting is categorised as a public health concern when it is above 20% (5). The prevalence of stunting was high, at 41.7% in this study far above the reported SADHS 2016 of 22% in Limpopo and 28.1% at national level and Limpopo province. The observations are thus worrisome as this municipality has higher stunting than the provincial prevalence. Phooko-Rabodiba *et al* (14) reported similarly high rate of stunting at 39.6% in Sekhukhune district for children below 60 months. According to the WHO classification more than 40% indicates a very high prevalence of stunting among children. Stunting reflects chronic undernutrition and is associated with an underdeveloped brain, with long-lasting destructive consequences, including diminished mental ability and learning capacity, poor school performance in childhood, reduced earnings and increased risks of nutrition related chronic diseases such as diabetes, hypertension, and obesity in future (2). The contributing factors in this study may have been diseases, nutrient intake, food insecurity, and sanitation, as caregivers' general nutrition knowledge and feeding practices were not found to be influencing factors (15). Correlation was only seen with water consumption and stunting, suggesting that water replaced nutritious foods. A study conducted in the same district revealed a high rate of unemployment, poor household income and purchasing power, and high level of food insecurity (14). Since stunting tends to have adverse and long-lasting consequences such as impaired cognitive ability and reduced productivity later in life (16; 17), there is a need to establish sustainable interventions to combat chronic undernutrition among this age group.

**Wasting**: None of the children in this study were found to be wasted. SADHS 2016 reported wasting prevalence of 4.7% in Limpopo. Wasting is a strong predictor of mortality among children under five. It is usually the result of acute significant food shortage and/or disease (11). The WHO (18) stated that, provided that there is no severe food shortage, the prevalence of wasting is usually below 5%, even in poor countries. Well researched strategies as stated in World Health Assembly, Global Nutrition Targets 2025: Wasting Policy Brief (19) need to be strengthened and implementation should be monitored to maintain a low prevalence of wasting. Such strategies include promotion of, and support for breastfeeding, nutrition counselling for families regarding complementary feeding practices, and the provision of food supplements. For older children, the focus should be on improving family foods (diversity, quality, and safety).

**Overweight and obesity:** In this study, 18.3% of the children were found to be overweight, compared to 13% reported at national level by SADHS 2016. The statement by the WHO reveals that the number of children who are overweight or obese in Africa has nearly doubled from 5.4 million in 1990 to 10.6 million in 2014 (19). Similar results, for the prevalence of overweight (18.1%), were reported in the SANHANES-1 in 2013, compared to the report by the NFCS study (10.6%) (13). According to the WHO overweight and obese children are more likely to develop non-communicable diseases such as diabetes and cardiovascular diseases at a younger age and are likely to stay obese into adulthood (19). Hence, childhood obesity is associated with a higher chance of premature death and disability in adulthood. At least 2.6 million people die each year as a result of being overweight or obese (19).

Overweight and obesity of caregivers co-existed with stunting in this study population. Most of the caregivers were classified as overweight to severely obese, while the stunting prevalence amongst children was high. This double burden of malnutrition is caused by inadequate pre-natal, infant, and child nutrition, which is then followed by exposure to high-fat, energy-dense, micronutrient-poor foods, and a lack of physical activity as the child grows older (19). A shift in diet towards increased intake of energy-dense foods, but low in vitamins and minerals, together with decreased physical activities, are some of the contributing factors towards overweight and obesity. Household food distribution, cultural practices, and a lack in variety of food may also have an effect on overweight and obesity prevalence. It is not uncommon to find under-nutrition and obesity existing side-by-side within the same country, the same community, or even within the same household (19). Bhutta et al. (20) suggests the promotion of appropriate complementary feeding practices, increasing dietary diversity, and providing multiple micronutrient supplements with iron, as possible methods to improve linear growth without contributing to overweight.

**Nutrition knowledge of caregivers**: Themes of the nutrition knowledge among the caregivers was mainly on early infant feeding, complementary feeding, consumption of a variety of nutritious food, number of meals to be given to young children, and the source of their nutrition information. The results of this study indicate that there is still a huge challenge regarding caregivers' nutrition knowledge on the benefits of exclusive breastfeeding and continued breastfeeding up to two years and beyond, also on the importance of timeous introduction of nutritious solid food to the children. Similar findings were reported where caregivers cited ceasing to provide breast milk to their children around one year of age because the milk was regarded as not nutritious after one year, and would instead switch to formula feeding in some cases or an entirely solid food diet in other cases (21). The main constraint to the timeous introduction of solid food (from six months of age) is the mother's lack of knowledge (22).

Regarding general nutrition knowledge, caregivers could not determine foods of the same food groups. For example, potatoes were regarded as the alternative for meat by some caregivers. Their knowledge could have been influenced by their traditional practices, as potatoes were mostly eaten with porridge as relish, as many consider it a vegetable. The mothers' nutrition knowledge was poor even on fruit consumption, meaning improvement in micronutrient deficiency would still be a challenge.

In this study some of the caregivers reported that they were not given any nutrition information, whereas about half of the caregivers reported health professionals as the source of nutrition education and a few cited media. The study done in Limpopo (23) reported that the majority of mothers (76%) said they had not been taught which foods were good for their babies, and only 13.5% said that they were informed by health workers or nurses. The quality of nutrition knowledge among health workers needs to be strengthened in order to provide high quality and consistent nutrition information to the caregivers.

**Feeding practices of children**: Almost all children had been breastfed at one stage in their lives, however, approximately half of the children were given infant formula while they were still less than six months. Introduction of solids foods before six months seemed to be a common practice. Similarly, in the study conducted in Vhembe district, Limpopo province (23), exclusive breastfeeding was reported to be only 7.6%, even though the prevalence of breastfeeding in general was reported to be at 97%. According to SANHANES-1, only 7% of children aged ≤ 6 months were exclusively breastfed at national level (13). Low rate of exclusive breastfeeding was also observed in a study undertaken in Malawi (24), where only 13.3% infants who were followed up from birth to twelve months were exclusively breastfed. Although breastfeeding has been a common practice amongst Africans, the WHO recommendation of exclusive breastfeeding for six months has not been complied with (5). SADHS however, observed that the proportion of infants under age 6 months who are exclusively breastfed increased from 7% in 1998 to 32% in 2016 in South Africa (12).

Children in the current study did not consume a diversified diet. Starchy foods were commonly consumed, with a low consumption of fruit, vegetables, animal proteins, and alternative protein sources. Very few children were given vegetables daily with only one out of eight children given fruit daily. Meat/meat products were given two to three days per week to half of the children. Similar findings were reported (25) where the same challenge of lack of variety in children's meals. Smuts et al. also reported that more than half of the children aged 0 to 71 months in rural districts of KwaZulu-Natal and the Eastern Cape seldom or never consumed meat products (26).

The top ten foods that were mostly given to the children were stiff porridge, bread, tomatoes, potatoes, potato/maize/corn snack chips, chicken heads and feet, chicken, orange, biscuits, and eggs. It has been reported that at national level, the most commonly consumed food items in South Africa were maize, sugar, tea, whole milk, and brown bread (27). SANHANES-1 indicated that the average South African diet of a child is energy dense, whilst micronutrient poor (13). The households in this study seemed to depend more on less expensive food that are energy dense. Households may be opting for these cheaper foods due to low socioeconomic status, as the majority were unemployed (14).

Low consumption of fruits and vegetables and protein may have adverse nutritional consequences with resulting micronutrient deficiencies such as iron, vitamin A and C, folate, and potassium, together with dietary fibre. All these nutrients are required for optimal growth and prevention of diseases. Low consumption of fruits, vegetables, and protein may be due to lack of access, unemployment, and lack of nutritional knowledge on the benefits and consequence of protein and micronutrient deficiencies.

**Influence of caregivers' nutrition knowledge and feeding practices on the nutritional status of the children**: Caregivers' nutrition knowledge and feeding practices were not found to influence nutritional status of children in this study. These findings were contrary to what was reported by other researchers, who indicated that a lack of awareness and a lack of nutrition knowledge regarding required feeding amounts, frequency of feeding, types of foods, and a balanced diet contribute significantly to poor nutritional status of children younger than five years of age, even in families where adults met their daily nutritional requirements (28). Nankumbi and Muliira (10) also reports that, in rural areas, primary caregivers were unable to use IYCFP to enhance the nutritional status of the children due to a lack of knowledge. Mushaphi et al. (23) concluded that caregivers' nutritional knowledge affects the way they feed their children and consequently affects the nutritional status of the children.

Stunting was positively correlated to household income and range of household income. In addition, weight-for-age was positively correlated with source of income. According to UNICEF when unemployment and low wages are presenting factors, families eat cheaper foods, which are less nutritious, leading to weight loss and malnutrition (11). This suggests that, to improve nutritional status in this population, interventions need to focus on improving the household income and thus food accessibility.

Although studies using a heterogeneous sample are necessary to explore possible relationships between knowledge, feeding practices, and nutritional status, this sample was homogenous in terms of sociodemographic and household parameters. Thus, their practices were likely to be similar. The sample also came from a rural setting with mainly households from villages that were underdeveloped. Nutrient intakes and levels of food security were not determined. Thus, the co-existence of stunting and overweight could not be fully explained.

In conclusion, nutrition knowledge of caregivers did not influence the feeding practices of children, however poor knowledge was observed. There is a serious need for appropriate nutrition education to capacitate caregivers to make proper food choices and be made aware of the importance of eating nutritious, indigenous foods. Improving economic growth in rural areas can generate more income, especially for caregivers, and may lead to improvements in health and nutrition status of young children.
